# More polished, not necessarily more learned: LLMs and perceived text quality in higher education

**DOI:** 10.3389/frai.2025.1653992

**Published:** 2025-12-01

**Authors:** Betty Tärning, Trond A. Tjøstheim, Annika Wallin

**Affiliations:** Department of Philosophy and Cognitive Science, Lund University, Lund, Sweden

**Keywords:** LLM, generative AI, higher education, student learning outcome, academic writing, text quality, peer assessment

## Abstract

The use of Large Language Models (LLMs) such as ChatGPT is a prominent topic in higher education, prompting debate over their educational impact. Studies on the effect of LLMs on learning in higher education often rely on self-reported data, leaving an opening for complimentary methodologies. This study contributes by analysing actual course grades as well as ratings by fellow students to investigate how LLMs can affect academic outcomes. We investigated whether using LLMs affected students’ learning by allowing them to choose one of three options for a written assignment: (1) composing the text without LLM assistance; (2) writing a first draft and using an LLM for revisions; or (3) generating a first draft with an LLM and then revising it themselves. Students’ learning was measured by their scores on a mid-course exam and final course grades. Additionally, we assessed how the students rate the quality of fellow students’ texts for each of the three conditions. Finally we examined how accurately fellow students could identify which LLM option (1–3) was used for a given text. Our results indicate only a weak effect of LLM use. However, writing a first draft and using an LLM for revisions compared favourably to the ‘no LLM’ baseline in terms of final grades. Ratings for fellow students’ texts was higher for texts created using option 3, specifically regarding how well-written they were judged to be. Regarding text classification, students most accurately predicted the ‘no LLM’ baseline, but were unable to identify texts that were generated by an LLM and then edited by a student at a rate better than chance.

## Introduction

That LLMs such as ChatGPT will change higher education has been clear since at least 2022. We can expect a permanent change in how students work and learn, as these tools are implemented in for example information search, studying, writing and programming. *How* they are implemented is, however, also likely to change their impact. It is our impression that a majority of teachers in higher education are worried by the emergence of easily accessible LLMs (Dwivedi et al., 2023; see Yigci et al., 2025). Not only do they make it easier for students to cheat, and impede teachers ability to detect cheating, but more importantly, students do not always train core academic competencies sufficiently to actually master them (Kosmyna et al., 2025; Jošt et al., 2024). In particular, increased reliance on these tools can entail that students receive less training in writing (with text produced by, or in cooperation with LLMs) (Deep and Chen, 2025; Kosmyna et al., 2025), in programming (with LLMs generating software codes based on prompts) (Jošt et al., 2024; Lyu et al., 2024) and in independent thinking (with material selected by and adapted to the relevant context by LLMs), (Deep and Chen, 2025). On the other hand, generative AI can also be used to enhance these and similar skills (Lyu et al., 2024). As students become more efficient (offloading tasks to relevant LLM-based systems) more time can be made available for other types of learning activities (Tasdelen and Bodemer, 2025). The possibility to get relevant feedback and information tailored to specific needs allows for personalized learning (Tasdelen and Bodemer, 2025; Lee and Moore, 2024). Also, end products of higher quality (no matter who produced them) have been found to affect both self-esteem and motivation (Tasdelen and Bodemer, 2025; Mohammed and Khalid, 2025). Regardless of whether we focus on the positive or negative aspects of LLMs in higher education, it is important to keep in mind that if we change how students think and learn when working academically, this will have important downstream effects on how, and how well, students write and think in general.

In any case, LLMs, including chatbots such as Chat GPT will not disappear in the near future. Banning them from academia altogether is not only practically impossible, but also likely counterproductive. Consequently, we need to know more about *how* LLMs affect student learning; when to counteract them, and how to best put them to use. In this paper we present a study investigating the consequences of using LLMs in a student population. In particular, we attempt to answer three questions. First, and most importantly: How does using LLMs in an assignment affect student learning as measured by performance on a written exam on the same topic, as well as on a longer final course assignment. Secondly, we ask how students rate texts in each of the three categories: (a) written by students on their own with no LLM; (b) written by a student but revised by an LLM; and (c) written by an LLM and approved by a student. That is, how is the (perceived) quality of actual student assignments affected by access to LLMs. Thirdly, can fellow students identify texts that have been written with the aid of LLMs?

### Background

Large language model (LLM) chatbots can proficiently mimic human-like communication – when prompted well, they can help with text-based tasks, like essay writing and code generation. However, we still do not know when and how these systems are beneficial to human learning and when they are detrimental: so far studies show mixed results.

In higher education, several authors have argued that LLMs can assist researchers and students with tasks such as text completion, language translation, and responding to academic queries (Peláez-Sánchez et al., 2024; Dwivedi et al., 2023; Kasneci et al., 2023; Lund et al., 2023). Other researchers point out that LLMs can provide students with immediate and personalized feedback which improves students’ understanding of new concepts (Pardos and Bhandari, 2023; Anders, 2023; Khan et al., 2023). Other researchers are concerned that generative AI may spread misinformation and provide inquirers with inaccurate explanations, (Liebrenz et al., 2023). Additional worries are reduced creativity and originality (Dwivedi et al., 2023), privacy risks and ethical issues (Chukwuere, 2023; Lund and Wang, 2023). A last important concern is the ever present risk of cheating – in particular on unsupervised exams. These worries are set against a background of broad technological gains in terms of LLM capabilities in, e.g., software programming (e.g., Hou and Ji, 2024; Peng et al., 2024) and image synthesis (e.g., Tan et al., 2023).

#### LLMs and learning

Successful learning depends on actively engaging with the learning material, in contrast to passively reading a text without spending effort (Willingham, 2023). Using an LLM-based chatbot can be engaging, but allows for passive reading without reflection. LLMs can encourage students to seek quick answers instead of engaging in the more demanding cognitive processes required for deep learning (Baker, 2019). This ambiguity between technology engagement and learning passivity means that it is unclear how LLMs will affect student learning going forward. For example, Swargiary (2024) presents results demonstrating how the use of ChatGPT among undergraduate students led to significant increases in cognitive engagement (measured by a self-report questionnaire). Unfortunately, a pre- and post-test – conducted by multiple-choice and short-answer questions – showed lowered academic performance compared to a control group that used traditional study methods. Interestingly, using the chatbot was associated with more self-reported procrastination, operationalised using the Procrastination Assessment Scale (Lay, 1986). Stadler et al. (2024) tasked participants with doing online research either with a traditional search engine or with ChatGPT. Using self-assessment of cognitive load, they found that participants using the LLM reported less cognitive load than those using a search engine. On the other hand, those using the search engine appeared to engage with the research with more depth - measured as the number and quality of relevant argument in their statements. A recent study by Kosmyna et al. (2025) indicates that the repeated use of chatGPT can negatively impact neural connectivity. During 4 months they let one group of participants use ChatGPT as their sole resource of information for an essay-writing task. Other groups could either use any website, but not ChatGPT, or were prohibited from using either. Participants were later asked to do a separate task without using neither websites nor ChatGPT. Interestingly, EEGs revealed that the ChatGPT group showed less coordinated neural efforts than the control group who had been prohibited from using ChatGPT or other websites during the first essay-writing task.

However, several studies – based on self reports – indicate that access to LLMs can enhance performance. In a study by Khan et al. (2024), students who report that they use LLMs for generating texts, seek assistance with assignments, or try to clarify course-relevant topics, also self-report higher academic performance. This is in line with the well reported fact that personalized learning improves academic achievement, engagement, and self-efficacy (Wu, 2017). Since LLMs such as ChatGPT can offer personalized assistance, there is a case for them improving learning (e.g., Javaid et al., 2023). Additionally, since students report increased motivation when they use LLMs (Caratiquit and Caratiquit, 2023), and motivation *can* be linked to performance (Cerasoli et al., 2014), there are some grounds for expecting improved student learning through LLM access.

Nonetheless, reasons to expect the opposite are apparent too. Common concerns about the effects of LLMs on learning include less training in critical thinking, and superficial information processing (Dwivedi et al., 2023; Lo et al., 2024). In addition, relying on someone else for providing answers (regardless of whether it is a peer or a chatbot) is likely to reduce the engagement required for learning to happen. This claim is backed by evidence that students using automated feedback tools often exhibit reduced cognitive effort and engagement with the learning material (measured by self-report questionnaires; Caratiquit and Caratiquit, 2023).

#### LLMs and writing

There are several ways in which LLM-based chatbots can help students complete written assignments. Most obviously, LLMs are useful for editing text, but they can also produce text independently (Graf and Bernardi, 2023). Additionally, chatbots can recommend books and articles relevant to specific topics (AlAfnan et al., 2023; Cooper, 2023; Fauzi et al., 2023). In the best of worlds, students would read and use these resources themselves. At the time of writing though, LLMs have limited ability to distinguish between reliable and unreliable sources. They are also prone to generating text which appears correct but is made up – an obvious problem for students with limited knowledge of a topic (Lee, 2024; Loh, 2023).

The most important problem with using LLMs to produce text goes beyond using false data. The act of translating thoughts into words is a way to actively sort out our thinking (Langer and Applebee, 1987). Hence, a real worry is that an individual using LLMs to produce text learns *differently* – and *less –* than students that produce text on their own. For instance, Noam Chomsky described using generative AI as “a way of avoiding learning” (Open Culture, 2023). There is, however, a difference between suspecting this outcome and actually observing it. In addition, there are different ways in which LLMs can be used to produce texts. One way is for students to ask LLMs for input on readability and structure for drafts they have written themselves. Another is for them to generate a text with an LLM and then edit and change it in ways that they find appropriate. But students may also simply generate text segments and leave it at that, after (hopefully) having read and approved of them. Depending on which method students use, they will be more or less engaged in producing the end result. It may even be that a student who generates text in collaboration with an LLM, revising back and forth, is more engaged in the text production than one who produces the entire text on their own.

#### Judging texts generated by LLMs

The quality of texts produced with LLMs – be it ChatGPT or other services – is now so high and humanlike that it can be difficult to tell the difference. For example, Gao et al. (2022) conducted a study in which they compared original abstracts (accepted to academic journals) with abstracts generated with ChatGPT. The AI was prompted with the original title and the name of the journal and required to produce an abstract. Human- and LLM-generated abstracts were then evaluated by an AI output detector, a plagiarism detector, and human reviewers. The human reviewers successfully identified 68% of the abstracts generated by an LLM, while misclassifying 14% of the human-generated abstracts as LLM-generated. The reviewers described that they judged superficial and vague abstracts as being generated by LLMs.

#### Summary

In summary, there are several ways in which using LLMs can help or hinder learning and academic development. The technology itself appears to be engaging, but this does not necessarily mean that students engage with the educational material in ways that increase learning. One of the main problems appears to be that using LLMs reduces the effort students spend on the material while spending effort is precisely what is required for effective learning to take place. However, there is also evidence that using an LLM as a personal tutor is efficient for learning. Tutoring in this context could be to explain difficult concepts and give advice adapted to students’ particular needs. With respect to writing and using LLMs to generate text, there are two major concerns. First, the propensity of LLMs to make up text that looks valid on the surface. Second, the process of creating text is intrinsically a way for students to improve their understanding of material and sort out their thinking. Hence, having text served to them by a machine is a lost learning opportunity. Finally, it is becoming harder for humans to distinguish generated text from text written by humans. A remaining worry is that the prevalence of self-reports in studies on LLM use in higher education, has created a need for complimentary methodology. This need is addressed here- We use both actual grading data, as well as judgements by fellow students to investigate whether using LLMs makes any difference to academic achievement. We also investigate what those differences might be in particular. The next section describes our research questions in more detail.

#### Research questions

In this paper we use the learning outcomes of computer science students - grades and mid-exam scores on an introductory course in cognitive science - to examine how successful different ways of dividing labour between LLM and student are. In particular we presented the students with three options: (1) no LLM (2) LLM Collaboration - writing writes a first draft and then using an LLM for revisions (3) LLM Approve - the LLM generates the text and the student approves it. The study has three research questions:

*RQ1*: How does use of LLMs and different divisions of labour between student and LLM (No LLM, LLM Collaboration, and LLM Approve) affect student learning as measured by (a) points received on a mid-course exam and (b) final course grades

*RQ2*: How are texts for each LLM option graded/rated by fellow students with respect to how well-written, easy to read, and how credible the text is

*RQ3*: Can students readily distinguish LLM-generated texts from student-generated texts?

## Method and material

The study was an integral part of a course in cognition held at a technical university in Sweden. Students were first year students in computer science. During this course students write an individual report, write a mid course exam on the same topics as the individual report and hand in a final written assignment, discussing the same topics on a more general level. The results on this written assignment, together with the mid course exam determine students’ grades (fail, 3, 4, 5, with 5 being the highest grade).

The individual report is an obligatory learning activity that students may base parts of their final written assignment on, and is presented to students as a first draft of the final assignment, that will receive feedback from a teaching assistant. The individual report is due the day before the mid course exam (in-class exam) that covers the same topics, and should be used as preparation for this exam. When writing the individual report, students were given three voluntary options: (i) to write it entirely on their own (“No LLM”), (ii) to write it in collaboration with a suitable LLM (e.g., ChatGPT, Claude, or Gemini) (“LLM Collaboration”) or to produce the text using an LLM and hand it in after having read and approved the text (“LLM Approve”). In other words, students were free to choose any LLM service they wanted, and no information about this choice was collected. It is important to note that students themselves selected which group they wanted to belong to. Students handed in their assignments online and separately reported which category of LLM-use they belonged to. They were informed that their teachers and teaching assistants would not be aware of this choice until the course had ended and all grades had been finalized. Students also indicated whether they wanted to be part of the study, after being informed that participation was voluntary, and, again, that teachers would not know if they had chosen to participate until all grades had been finalized. The study was approved by the Swedish Ethics Board (Dnr 2024–05394-01). For information given to students, with an English translation, see Appendix B.

### Participants

In total the course had 166 students and out of these, 118 students chose to participate in the study (26 = women and 92 = men). Five were removed for the mid-course exam analysis (due to not completing the exam) four out of these five were removed from the final grade analysis (due to not completing the course).

### Measures

As part of the curriculum each student was required to read three other individual reports and rate their quality (see below) and how they believed the texts had been produced. For students that agreed to take part in the study, the ratings their individual report had received, as well as points on the mid course exams and final grade (heavily based on students’ final assignment) were collected and used as dependent variables.

### Individual report ratings

The individual report is the first step in producing the final assignment that determines students’ course grade. It is handed in 17 days before the final deadline, and comprises a draft of 2–3 pages in which a particular technical interface is evaluated using relevant concepts from the course literature. This draft receives feedback from a teaching assistant, and was this year also anonymized and rated by fellow students. According to a randomized schedule, three anonymised texts were sent to each student. No student knew in what way the texts they had been assigned had been produced. Each student rated the three texts on how well written the text was, if the author appeared to know what s/he was talking about, if the author was trustworthy, and the overall quality of the text. Answers were given on a scale from 1 to 5, where 5 was the highest score. In addition students were asked how they thought the text had been produced, that is, exclusively by a student, in collaboration with an LLM or produced by an LLM and revised by a student. Full questions, translated to English can be found in Appendix C.

### Mid course exam

The day after the first individual report was due, students wrote a 2 h mid course exam, testing their understanding of central concepts (the same concepts that were used to analyse the technical interface in the individual report). Students could receive at maximum 26 points on the exam, and 15 were required to pass. The mean points for all students taking the course was 18.70 (SD = 2.85) and the students that agreed to take part in this study on average received 18.73 points (SD = 2.50). The exam consisted of 10 questions altogether (sometimes with multiple sub questions). In the last question (question 10) students were asked to analyze a technical interface using the concepts learned in the course. This particular question resembled the final assignments and was worth 5 points. Because of its resemblance to the final assignment the score for this last question was taken into account as a specific analysis. The mid course exam, translated to English can be found in Appendix D.

### Final assignment

The final assignment was divided into two parts, a mandatory part at which students could only pass (i.e grade 3) or not pass. Then there was an optional part which students themselves could choose to write to potentially receive a higher grade than 3 (i.e grade 4 or 5). This final assignment landed somewhere around 3,500 words (for the mandatory part) and around 1,300 words (for the optional part). For a translation of the final assignment into English, see Appendix E.

Taken together, scores/grades collected for analysis were; how students judged each others’ texts, points on written exam, points for last task (question nr 10) in written exam and final course-grade.

## Results

### Analysis

Bayesian statistical analysis was performed in R v4.5.0 using the brms package v2.22.0. We fitted two models to each of the outcome measures of the data, running each model for 10,000 iterations with 5,000 warmup iterations:


model2:outcome~ai_condition+(1∣subject)

model1:outcome~ai_condition

Here, outcome is one of *final_grade, competency, quiz_score, well_written, stars, quiz_final_part_score, easy_to_read, correct_concepts*, and *ai_condition* is one of No LLM, LLM Collaboration, or LLM Approve;, and subject is one of the set of codes for each student subject. Models were compared using leave-one-out cross-validation via loo_compare in R. The best fitting model was model 2 for all outcomes (see Appendix Table A1), and was used to predict mean and median values with 95% credible intervals.

*RQ1*: How does use of LLMs and different divisions of labour between student and LLM (student-generated, student-LLM collaboration, LLM-generated, student approved) affect student learning as measured by (a) points received on a mid-course exam and (b) final course grades.

The analysis for points of the mid-exam was based on the number of points the student received the first time they took the exam.

**Figure 1:**
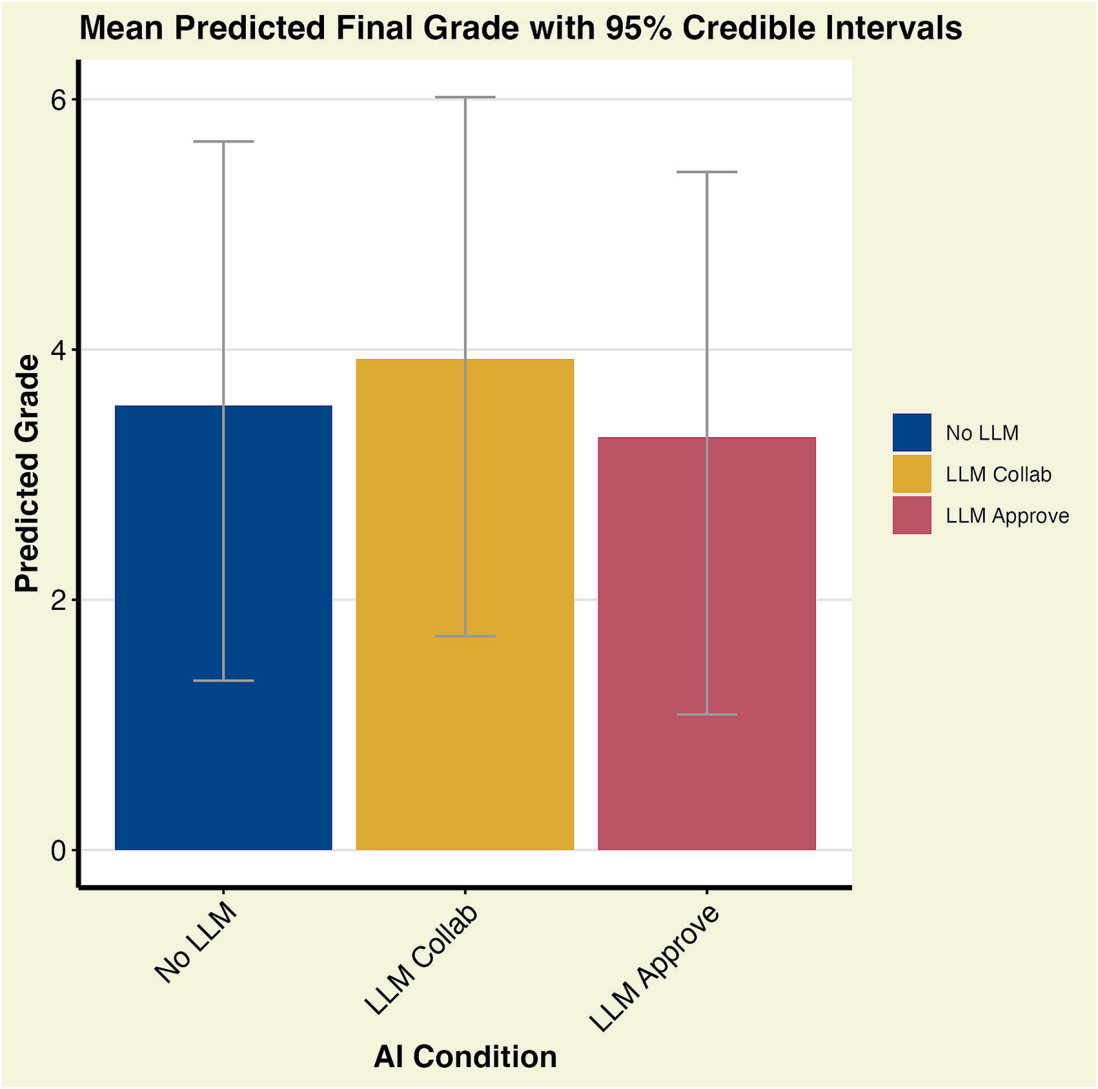
Final course grade mean values with 95% CI. The estimated difference between the No LLM baseline and the LLM Collab option is 0.37 with 95% CI [−0.15, 0.94] (see Appendix Table A3). Since the CI interval crosses zero, the evidence that LLM Collab improves grades is inconclusive.

**Figure 2:**
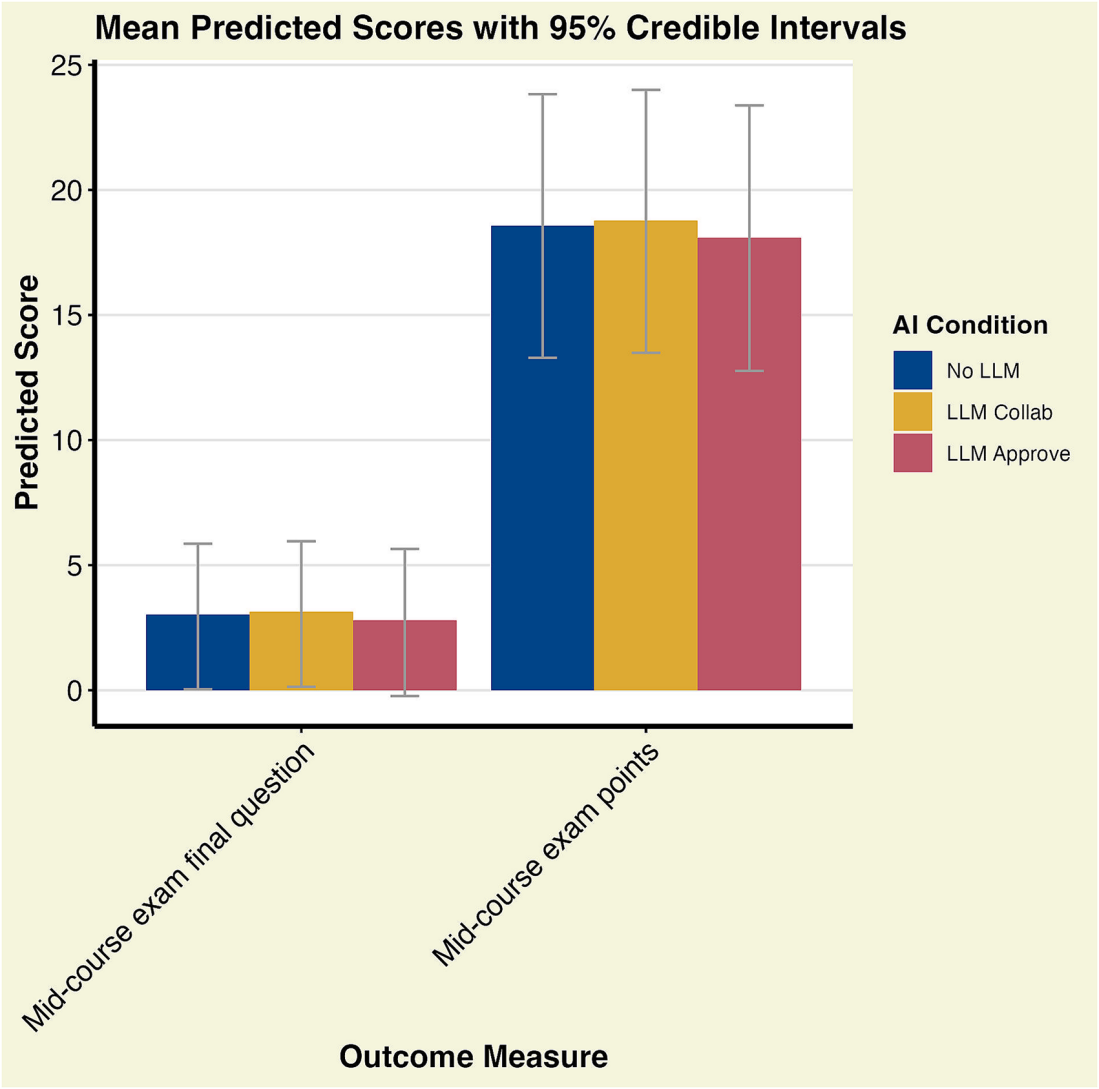
Predicted mean with 95% CI scores for final part of mid-course exam, and the total score for the mid-course exam.

For RQ1 we found no notable differences between our three groups in connection to score on mid-exam. For the final grade, the No LLM baseline had an estimated intercept of 3.54 and 95% cI of [3.08, 3.94]. The difference between the baseline and the LLM Collaboration option was 0.37 with a 95% CI of [−0.15, 0.94] indicating a weak effect, while the difference between baseline and the LLM Approve option was −0.25 with 95% CI of [−0.87, 0.35] (see Figure 1; Table 1; Appendix Table A3). For the mid-course exam where the students were required to sit in a university hall with exam guards, there are no notable differences between the LLM conditions (see Figure 2; Table 1; Appendix Table A3).

**Table 1:** Predicted mean and median values with 95% CI for final exam grade, scores for mid-course exam points, and for the final part of the mid-course exam.

LLM condition	Mean	Median	95% Credible interval of mean
Final grade			
No LLM	3.55	3.56	[1.35, 5.66]
LLM collaboration	3.92	3.93	[1.71, 6.02]
LLM approve	3.30	3.31	[1.08, 5.42]
Mid-course exam score			
No LLM	18.58	18.59	[13.29, 23.83]
LLM collaboration	18.77	18.79	[13.49, 24.00]
LLM approve	18.09	18.11	[12.77, 23.38]
Mid-course exam final part score			
No LLM	3.03	3.07	[0.04, 5.86]
LLM collaboration	3.13	3.17	[0.14, 5.95]
LLM approve	2.79	2.83	[−0.23, 5.65]

To see the distribution between students who chose to write the optional part and whether it differed between the different groups, we looked at the numbers descriptively. We could see that most students in the LLM Collaboration condition choose to write the optional part and fewest students in the LLM Approve condition (see Table 2). Recall that completing the optional part opened up the possibility for students of achieving the two highest scores – 4 and 5 – while the maximum score would otherwise be 3.

**Table 2:** Distribution of grades per LLM condition for the voluntary part of the final assignment.

LLM condition	Final grade	*n*	%
No LLM	4	5	35.7
	5	9	64.3
LLM collaboration	4	10	33.3
	5	20	66.7
LLM approve	4	4	57.1
	5	3	42.9

*RQ2*: How are texts, written with or without generative AI, graded/rated by fellow students with respect to how well-written, easy to read and how credible the text is

**Figure 3:**
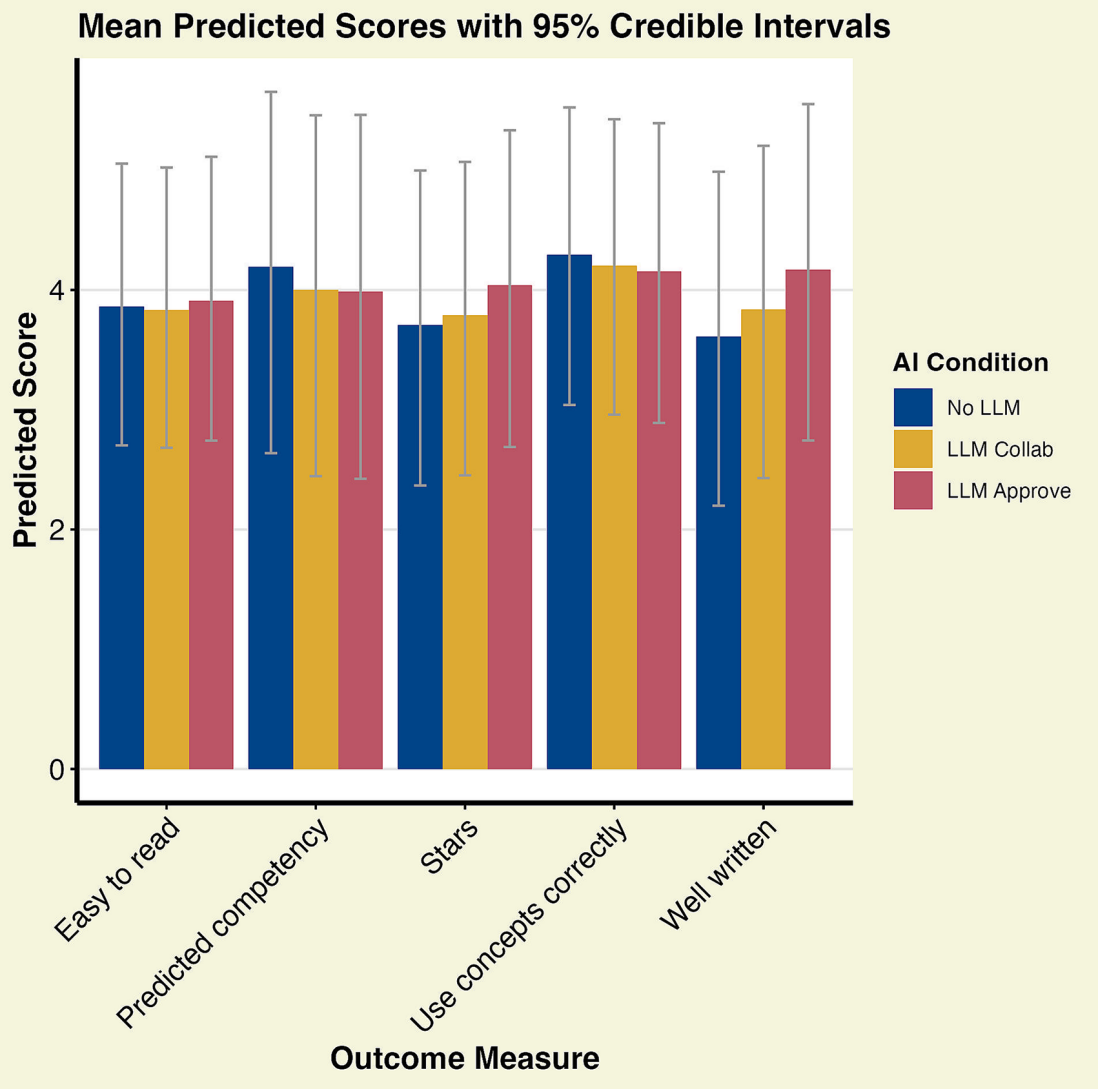
Student assessment scores. The “well written” score differs from the No LLM baseline with a 95% CI of [0.17, 0.97], while other scores have baseline differences with 95% CI that cross zero (see Appendix Table A3).

When it comes to grading the different texts we found a positive difference for the “Well written” assessment outcome, where the contrast LLM Approve vs. No LLM was estimated 0.55 with SE 0.20 and CI [0.17, 0.97]; the baseline No LLM Intercept was here 3.62 with SE 0.12, and CI [3.38, 3.85]. None of the other assessment outcomes had any notable contrasts (see Figure 3; Appendix Tables A2, A3).

*RQ3*: Can AI-generated texts be reliably distinguished from student-generated texts?

As a final question we looked at students’ ability to distinguish texts written with LLMs or not. Students were tasked with predicting the chosen LLM option of their assignees when assessing their mid-course work. The associated confusion matrix is shown in Table 3. The No LLM option was most commonly predicted correctly at 66.6%, while only 33.8% could predict the LLM Approve condition correctly. The highest end grades were found for the actual LLM Collaboration, predicted No LLM condition with mean grade 4.03 (SD = 1.18), which accounted for 48.6% of cases; this was also the most common prediction error. The lowest grades were for the actual and predicted LLM Approve condition at mean 3.14 (SD = 0.94), again accounting for 33.8% of cases.

**Table 3:** Confusion matrix for student’s predicted vs. actual LLM category with mean, SD final grade, indicating that students could recognise the no LLM condition most, but the LLM approve condition least accurately.

Actual condition	Predicted condition		
	No LLM	LLM collaboration	LLM approve
No LLM	**66.3% (63)**	23.2% (22)	10.5% (10)
LLM collaboration	48.6% (71)	37.7% (55)	13.7% (20)
LLM approve	32.3% (21)	33.8% (22)	**33.8% (22)**

Each student’s work was assessed by several co-students.Bold values indicate most and least accurately predicted condition.

## Discussion

This paper explored how LLMs affect student learning. More specifically, we investigated how different use of LLMs affect student performance (as measured by points received on a mid-course exam and final course grades), how co-students rate fellow students texts’ quality, and whether it is possible for students to identify if texts have been written with the aid of LLMs or not. From the results we can see that the effects of using LLMs are not that clear – given overlapping intervals from the Bayesian model. Although fellow students apparently grade texts written with the aid of LLMs (group 3 “LLM Approve”) higher than the two other conditions texts, we caution that any distinction is uncertain and may reflect sampling variability rather than a true effect. Similarly, the other two research questions gave unclear results too.

Concerning RQ1, whether the use of LLMs affects student’s learning as measured by their final grade, we found a weak differences between the baseline and the LLM Collaboration condition. Our results hence somewhat align with Khan et al. (2024) or Javaid et al. (2023). In particular the results show that the LLM Collaborate condition had a higher mean final course grade score than the other two conditions, though the overlapping CI interval means that more precision is needed to draw a conclusion. Looking at the distribution among students writing the optional part we could see that 30 (out of 51 students) belonged to the LLM Collaboration condition and 14 students to the No LLM condition and only 7 students belonged to the LLM Approve condition. Speculating, this could be because of the possibly significant time and effort savings LLMs can afford.

However, this goes against the fact that only 7 students chose to write the optional part in the LLM Approve condition. It might be that students belonging to this condition (LLM Approve) are students who do not prioritize this particular course. It is an obligatory course but lies outside the student’s core program. This may have affected how much effort they spend on the course and hence may be willing to make savings with LLMS. The number of students who received grade 3 was quite high (only three of students did not pass the final written assignment). The high number of students who passed the exam could be traced to the fact that students handed in the individual report in the middle of the course and received some feedback from their teacher assistant. But most of all, the fact that so many students passed was probably because they were forced to start with the final assignment well before it was due.

Some further remarks on the optional part relative to the overall scoring can be made by observing that more ambitious students (or at least those motivated to put in extra work) tend to complete it. It is plausible that students that are interested in the course and want to secure the best score possible also are competent users of LLMs in terms of effective prompting, meaning that they can employ it well in both the LLM Collaborate and LLM Approve conditions. Being allowed to use a chatbot for the course might also have lowered the threshold for attempting the optional part, such that students that otherwise would not have tried did so. However, no conclusions about these speculations can be drawn from the Bayesian analysis, and more insight must wait until addressed by a targeted study. To this we should add that students themselves selected whether and to what extent they used LLMs. Hence, if more ambitious students select the LLM options, this will in itself explain this result.

We also looked at the final question for the mid-course exam and whether we could find any differences between conditions here. Mean values show students in the full LLM condition scoring lower on both the mid-course exam and the final course grade and students in the LLM Collaborate condition scoring the highest. However, the 95% CI of the differences crosses the zero point, preventing any meaningful conclusions. A possible explanation of the result is that it is produced by differences between the students that volunteered for the different conditions (level of LLM-use). Ideally we should have randomly assigned students to each condition, but this was not possible both from a student rights perspective and for practical reasons. Since the study was an integral part of an already existing course, the study was required to stay within that course’s framework. Moreover, we felt it was unethical to compel students to use learning methods that we considered less effective than available alternatives.

In RQ2 we asked, *how are texts, written with or without LLMs, graded/rated by fellow students with respect to how well-written, easy to read and how credible the text is.* Here, students scored texts which were produced with the LLM Approve option higher in terms of how well written they were than those in the No LLM condition (CI 2.74–5.55). A reason for this might be that LLMs are quite good at producing well formed, grammatically correct sentences without spelling mistakes. They were after all first conceived of as translation tools. Our results are in line with previous research suggesting that LLMs can effectively be used to write text and improve scientific language (Graf and Bernardi, 2023; Kasneci et al., 2023; Dwivedi et al., 2023). Writing essay-like text like those students wrote in this study are also where LLMs tend to shine.

A similar but weaker pattern showed how many “stars” - an overall grade - was assigned by students to a text. Again the LLM Approve condition had the highest mean score, even if the wide overlapping credible intervals necessitates future studies to be able to draw any conclusions. This qualification holds also for the scoring categories “correct concepts” and “competency,” where the No LLM condition has higher mean scores. Looking at these four together, and taking into account that students were most accurate in recognising the No LLM condition, it could be that a less perfectly written text was used as a reliable predictor of human rather than machine origin. As a consequence it is more plausible that the person behind the text indeed is more competent and also has understood concepts sufficiently to use them correctly. Still, the apparent flawlessness of the LLM produced text may have biased students towards giving these texts the highest overall number of stars.

Another explanation for why our categories did not yield larger contrasts is the fact that we cannot know if the students assessed the texts from the viewpoint of being a text written with or without help from LLM. That is, if they from beforehand (or quite early on) assess a text as written without the help from an LLM their judgments on how well-written and how fluent the text is might have been influenced. But, these premises were the same for all students and hence should not have affected the overall judgements.

This leads us to RQ3, *can AI-generated texts be reliably distinguished from student-generated texts?* Recall that for assessing texts written by the LLM collaborate group almost half of these texts (48.5%) were categorised as texts written with No LLM. 37.7% of the texts were however categorised correctly, that is, as texts edited with LLM. The hardest texts to categorise correctly were texts written in the LLM Approved category where the correct categorization of text did not differ from chance (see Table 3). Why might this be so? First, the LLM Collaboration condition could be “less perfect” than the LLM Approve condition; i.e. the editing process could introduce human errors that the students latched onto, making them misclassify these texts. Second, the almost perfectly flat distribution for the LLM Approve condition might simply indicate that students have insufficient experience in recognising LLM-written texts, making them uncertain of whether a well written text is due to a proficient student or to an LLM. On the one hand, these results do not align with what Gao et al. (2022) found, i.e., that humans were good at detecting abstracts generated by an LLM. But on the other hand, the compared texts were very different.

Overall, the absence of strong differences between the conditions is itself interesting because it indicates that using LLMs makes little difference in either direction except for how well written (linguistically) a text is perceived. Hence in the context of this particular study, LLMs appear to yield benefits as a form of advanced autocorrect, but do not affect the actual content of the text very much. Further on, even if there was a weak and uncertain effect for the “predicted competency” (estimated effect −0.21 [−0.61, 0.23] compared to No LLM baseline, Appendix Table A3) and “use concepts correctly” (estimated effect −0.14 [−0.48, 0.21] compared to No LLM baseline, Appendix Table A3), our analysis could detect little difference between the LLM conditions. We also acknowledge the limitation of operationalising outcomes as measured scores, which leaves the question of actual learning open. A future study could thus combine grades and scores with questionnaires about study material to possibly gauge the learning aspect more precisely.

To improve future research, we recommend adding a fourth option for students: a statement indicating whether they simply copied and pasted content directly from an LLM with minimal or no editing. In this study, the third option involved students using an LLM to generate a draft of their paper, which they then revised. However, we lack direct measures to confirm whether students actually processed the LLM output or simply used it without any changes. Following the study, the program director shared that students were explicitly informed from the beginning that using LLMs constitutes cheating. However, if LLMs were used, they were required to disclose how and where they were employed. This information was also communicated from students through representatives during the course evaluation. Based on this, we can reasonably assume that students did, in fact, actively revise any LLM generated text. The lack of performance differences (grades, mid-exam scores) between conditions might therefore be attributed to the consistent involvement of the students in all scenarios.

The current study allowed students to choose their LLM usage, which introduced self-selection bias into the data. Future research could mitigate this by randomly assigning students to groups. Additionally, a limitation regarding rating of fellow students’ texts is that not all texts were assessed by an equal number of students, potentially leading to unbalanced scores for some. While texts were randomly distributed to ensure each student reviewed one from each of the three options, this ideal was not always achieved due to the self-selected nature of the LLM options. We would like to emphasise again that this study contributes to the discussion regarding the use of LLMs in higher education by complimenting extant self-report data with peer rating scores and actual course grading outcomes. For better or worse, our data indicate that using LLMs make no certain difference to the measures we employed – although text quality may have been polished, academic outcomes were not affected in either direction.

As a final remark, we note that a key objective of the course during which data collection was done, is to enhance students’ writing skills. Beyond any insights about LLM use, the course evaluation reveals an urgent need for more writing practice, as evidenced by the consistently low ratings of text quality.

## Data Availability

The raw data supporting the conclusions of this article will be made available by the authors, without undue reservation.
