# Supplementary material for: More polished, not necessarily more learned: LLMs and perceived text quality in higher education
Source: Front Artif Intell. 2025 Dec 1;8:1653992. doi: 10.3389/frai.2025.1653992 (PMC12702930; doi:10.3389/frai.2025.1653992)
Supplement: Supplementary file 1 [file Supplementary_file_1.docx]

# Appendix A

**Table A1:** Model comparison, showing that hierarchical model predicts data best for all outcomes

| **Outcome** | **Type of Model** | **elpd_diff** | **se_diff** |
| --- | --- | --- | --- |
| Final grade | Model 2 | 0.0 | 0.0 |
|  | Model 1 | -8.2 | 1.4 |
| Mid-course exam score | Model 2 | 0.0 | 0.0 |
|  | Model 1 | -7.9 | 0.8 |
| Mid-course exam final part score | Model 2 | 0.0 | 0.0 |
|  | Model 1 | -12.3 | 1.4 |
| Easy to read | Model 2 | 0.0 | 0.0 |
|  | Model 1 | -8.5 | 1.2 |
| Competency | Model 2 | 0.0 | 0.0 |
|  | Model 1 | -4.6 | 1.0 |
| Correct concepts | Model 2 | 0.0 | 0.0 |
|  | Model 1 | -0.8 | 0.6 |
| Stars | Model 2 | 0.0 | 0.0 |
|  | Model 1 | -10.4 | 1.0 |
| Well written | Model 2 | 0.0 | 0.0 |
|  | Model 1 | -7.8 | 0.8 |

**Table A2**: Mean predicted scores for individual reports

| **LLM Condition** | **Mean** | **Median** | **95% Credible Interval of Mean** |
| --- | --- | --- | --- |
| Easy to read |  |  |  |
| No LLM | 3.86 | 3.86 | [2.70, 5.06] |
| LLM Collaboration | 3.83 | 3.83 | [2.68, 5.02] |
| LLM Approve | 3.91 | 3.90 | [2.74, 5.11] |
| Predicted competency |  |  |  |
| No LLM | 4.19 | 4.21 | [2.64, 5.65] |
| LLM Collaboration | 4.00 | 4.02 | [2.45, 5.46] |
| LLM Approve | 3.99 | 4.00 | [2.42, 5.46] |
| Stars |  |  |  |
| No LLM | 3.71 | 3.72 | [2.37, 5.00] |
| LLM Collaboration | 3.79 | 3.80 | [2.45, 5.07] |
| LLM Approve | 4.04 | 4.05 | [2.69, 5.33] |
| Use concepts correctly |  |  |  |
| No LLM | 4.29 | 4.30 | [3.04, 5.52] |
| LLM Collaboration | 4.20 | 4.21 | [2.96, 5.43] |
| LLM Approve | 4.15 | 4.16 | [2.89, 5.39] |
| Well written |  |  |  |
| No LLM | 3.61 | 3.62 | [2.20, 4.99] |
| LLM Collaboration | 3.84 | 3.85 | [2.43, 5.20] |
| LLM Approve | 4.17 | 4.18 | [2.74, 5.55] |

**Table A3**: Model parameters for best fitting model

| **Outcome** | **Parameter** | **Estimate** | **Std. Error** | **95% Credible Interval** |
| --- | --- | --- | --- | --- |
| Final grade | Intercept (Baseline: No LLM) | 3.54 | 0.22 | [3.08, 3.94] |
|  | Effect: LLM Collaboration vs. No LLM | 0.37 | 0.29 | [-0.15, 0.94] |
|  | Effect: LLM Approve vs. No LLM | -0.25 | 0.32 | [-0.87, 0.35] |
|  | SD (Code Intercepts) | 0.62 | 0.32 | [0.04, 1.11] |
|  | Residual SD (Sigma) | 0.81 | 0.23 | [0.42, 1.17] |
| Mid-course exam score | Intercept (Baseline: No LLM) | 18.58 | 0.45 | [17.65, 19.45] |
|  | Effect: LLM Collaboration vs. No LLM | 0.19 | 0.60 | [-0.96, 1.45] |
|  | Effect: LLM Approve vs. No LLM | -0.49 | 0.72 | [-1.89, 0.93] |
|  | SD (Code Intercepts) | 1.39 | 0.77 | [0.08, 2.66] |
|  | Residual SD (Sigma) | 2.06 | 0.62 | [0.58, 2.91] |
| Mid-course exam final part score | Intercept (Baseline: No LLM) | 3.03 | 0.24 | [2.55, 3.51] |
|  | Effect: LLM Collaboration vs. No LLM | 0.09 | 0.31 | [-0.51, 0.74] |
|  | Effect: LLM Approve vs. No LLM | -0.25 | 0.39 | [-1.02, 0.51] |
|  | SD (Code Intercepts) | 0.88 | 0.49 | [0.04, 1.73] |
|  | Residual SD (Sigma) | 1.07 | 0.40 | [0.23, 1.62] |
| Easy to read | Intercept (Baseline: No LLM) | 3.86 | 0.11 | [3.65, 4.07] |
|  | Effect: LLM Collaboration vs. No LLM | -0.03 | 0.14 | [-0.30, 0.24] |
|  | Effect: LLM Approve vs. No LLM | 0.05 | 0.17 | [-0.28, 0.39] |
|  | SD (Code Intercepts) | 0.34 | 0.18 | [0.02, 0.62] |
|  | Residual SD (Sigma) | 0.44 | 0.14 | [0.12, 0.64] |
|  |  |  |  |  |
|  |  |  |  |  |
|  |  |  |  |  |
| Predicted competency | Intercept (Baseline: No LLM) | 4.19 | 0.14 | [3.93, 4.46] |
|  | Effect: LLM Collaboration vs. No LLM | -0.19 | 0.19 | [-0.54, 0.19] |
|  | Effect: LLM Approve vs. No LLM | -0.21 | 0.21 | [-0.61, 0.23] |
|  | SD (Code Intercepts) | 0.40 | 0.21 | [0.02, 0.73] |
|  | Residual SD (Sigma) | 0.59 | 0.15 | [0.31, 0.83] |
| Stars | Intercept (Baseline: No LLM) | 3.71 | 0.12 | [3.49, 3.95] |
|  | Effect: LLM Collaboration vs. No LLM | 0.08 | 0.15 | [-0.24, 0.38] |
|  | Effect: LLM Approve vs. No LLM | 0.33 | 0.18 | [-0.03, 0.68] |
|  | SD (Code Intercepts) | 0.40 | 0.19 | [0.03, 0.69] |
|  | Residual SD (Sigma) | 0.48 | 0.16 | [0.14, 0.72] |
| Use concepts correctly | Intercept (Baseline: No LLM) | 4.29 | 0.12 | [4.07, 4.51] |
|  | Effect: LLM Collaboration vs. No LLM | -0.09 | 0.15 | [-0.40, 0.21] |
|  | Effect: LLM Approve vs. No LLM | -0.14 | 0.18 | [-0.48, 0.21] |
|  | SD (Code Intercepts) | 0.26 | 0.15 | [0.01, 0.53] |
|  | Residual SD (Sigma) | 0.54 | 0.09 | [0.36, 0.68] |
| Well written | Intercept (Baseline: No LLM) | 3.62 | 0.12 | [3.38, 3.85] |
|  | Effect: LLM Collaboration vs. No LLM | 0.23 | 0.16 | [-0.09, 0.54] |
|  | **Effect: LLM Approve vs. No LLM** | **0.55** | **0.20** | **[0.17, 0.97]** |
|  | SD (Code Intercepts) | 0.40 | 0.20 | [0.02, 0.71] |
|  | Residual SD (Sigma) | 0.52 | 0.16 | [0.20, 0.76] |

# Appendix B

**Information about the study "How does the use of generative AI affect learning at a higher level?"**

During the course, we will investigate how generative AI affects students' writing and learning. As teachers, we would like to reuse parts of what we discover in our research. This means compiling information from students who want to participate in the research study and presenting this in scientific contexts, such as in an article or at a conference.

**Confidentiality**

The information will be completely anonymized and only presented at group level (such as averages, correlations, and so on). The study is approved by the Swedish Ethical Review Authority (Dnr 2024-05394-01) and will be stored in accordance with Lund University's guidelines. Other researchers may access the anonymized information to ensure open research.

**Voluntary Participation and Procedure**

To ensure that no student feels compelled to participate in the research study, a person completely unrelated to the course will handle any consents to participate in the research. These will be stored without our access for one year. This person will compile the information we need and give it to the study leader (i.e., Betty Tärning) in anonymized form. Your teachers will never know whether you chose to participate in the research study or not. Those who participate in the study do not perform any additional tasks; the consent is about us using the anonymized information about you in our research. Students who choose not to participate will engage with the course in exactly the same way as participating students. Your participation is entirely voluntary, and you can withdraw at any time without explanation by contacting (blinded) (third-party email). This information will also be available on the course platform Canvas if you want to refer back to it. The results will be briefly presented in one of the course lectures, but you can also contact (blinded) to access the full study.

The information compiled by the person unrelated to the course or you includes:

- How you chose to approach your work in relation to generative AI
- The assessment your submission of "1.1 of the individual assignment" received
- The score you received on the quiz
- The score you received on the final assignment

**Risks**

We want to emphasize that as teachers/researchers, we will never know who chooses to participate in the study since all data and consent forms will be handled by a third party (blinded). Participation is entirely voluntary. However, as teachers, we will have access to the same information to grade.

The main person responsible for the study is:

Blinded

#

#

#

#

#

#

#

#

#

#

#

# Appendix C

**Text Assessment EXTA65**

Your task is to assess three different texts. To do this, you need to fill in the same link three times (after assessing text 1, click on "submit another response" at the bottom of the page to assess text 2 and text 3 respectively). Remember to fill in the correct "code" for the text you are assessing for each assessment round.

Your name (first and last name) ____________________________________

The text I am currently assessing has the code (e.g., blinded_to_read1.pdf). The most important thing is that the last number matches the file you are reading.

How well-written is the text? (How is the language?)

Not well-written at al Very well-written

1 2 3 4 5

How easy to read is the text? (Is there a flow in the text?)

Not easy to read at al Very easy to read

1 2 3 4 5

Does the author use concepts and theories from the course correctly?

I assess that the text I assess that the text

is completely incorrect is completely correct

1 2 3 4 5

Does it feel like the author knows what they are talking about? (Are they trustworthy?)

Not trustworthy at all Very trustworthy

1 2 3 4 5

In what way do you think the text is written?

1. Written entirely by oneself without AI.

1. Draft written by oneself - revised with AI.

1. Draft written with AI - revised by oneself.

How many stars would you give this text? (What is your overall impression of the text?) The more stars, the better the text.

⭐ ⭐ ⭐ ⭐ ⭐

1 2 3 4 5

# Appendix D

Exam in Cognition, EXTA65

Question 1 (max 4 p)

Below is a description of a digital chess game for beginners. Your task is to relate the various functions and features of the chess game to Norman’s concepts: constraints, affordances, signifiers, mapping, visibility, discoverability, feedback, mental/conceptual model, system image.

Choose the concept that best fits each item in the table below. A concept may appear more than once, and not all concepts may be relevant. Note that not all concepts necessarily apply in this context.


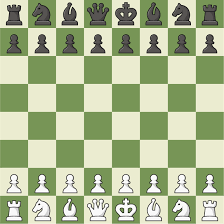


| Description | Concept |
| --- | --- |
| 1. The chessboard is a standard 8x8 board, and pieces cannot be moved outside the board. |  |
| 2. Pieces can only be moved according to the rules of chess. |  |
| 3. If the user hovers over a piece, arrows appear on the board indicating possible moves. |  |
| 4. The user can move pieces either by clicking and dragging them to the correct spot or using keyboard commands. |  |
| 5. Keyboard commands: To move a piece located at B4, for example, the user presses B + 4 and then uses arrow keys to move it. |  |
| 6. The piece being interacted with (by the user or the computer) lights up. |  |
| 7. The user can undo a move using the undo button or the Ctrl+Z command. |  |
| 8. All chess moves are displayed on the screen in a list. |  |
| 9. Chess moves are rated on a scale from 1–5 and displayed on the screen along with the moves. |  |
| 10. A complete manual for the game is available in PDF format and can be downloaded by the user. |  |

Question 2 (max 2 p)

Do the functions of the digital chess game align with a user’s mental/conceptual model of a real chess game? Choose two items from the table above – one that aligns with such a model and one that does not.

Is this alignment or discrepancy an example of good or poor design? Explain and justify your answers for both examples.

Question 3 (max 3 p)

Norman’s seven-stage model can be used to compare different solutions.
Below is a list of actions in randomized order. Number them according to the seven-stage model and name each stage.

1. I interpret what I see on the screen; I see that it says LU has about 47,000 students.
2. To Google, I need to open my computer, open the browser, go to Google, type my query, press enter, and wait.

3. I wonder how many students there are at LU and decide to find out.

4. I see on the screen that it says LU has about 47 000 students.

5. I actually perform the actions I decided to do (open computer, open browser, go to Google,
 type query, press enter and wait).

6. I evaluate whether what´s on the screen answers my original question (yes or no).

7. I could ask someone, call a friend who knows a lot, or Google. I decide to Google.

| Action (step number | Stage name according to Norman |
| --- | --- |
|  |  |
|  |  |
|  |  |
|  |  |
|  |  |
|  |  |
|  |  |

Question 4 (max 2 p)

In *The Design of Everyday Things*, Donald Norman describes “the gulf of execution” as one of two gulfs that can arise between a user and a system. The other is “the gulf of evaluation.”

Describe how one can help the user bridge “the gulf of execution” and “the gulf of evaluation.”

Question 5 (max 3 p)

In cognitive science, two types of attention processes are often discussed that describe how the brain receives and processes information: bottom-up and top-down.

Imagine you are buying a soda from a vending machine. How are bottom-up and top-down processes used in this situation?

Question 6 (max 2 p)

In what way does this website follow the Gestalt laws? Choose two laws and discuss based on them.


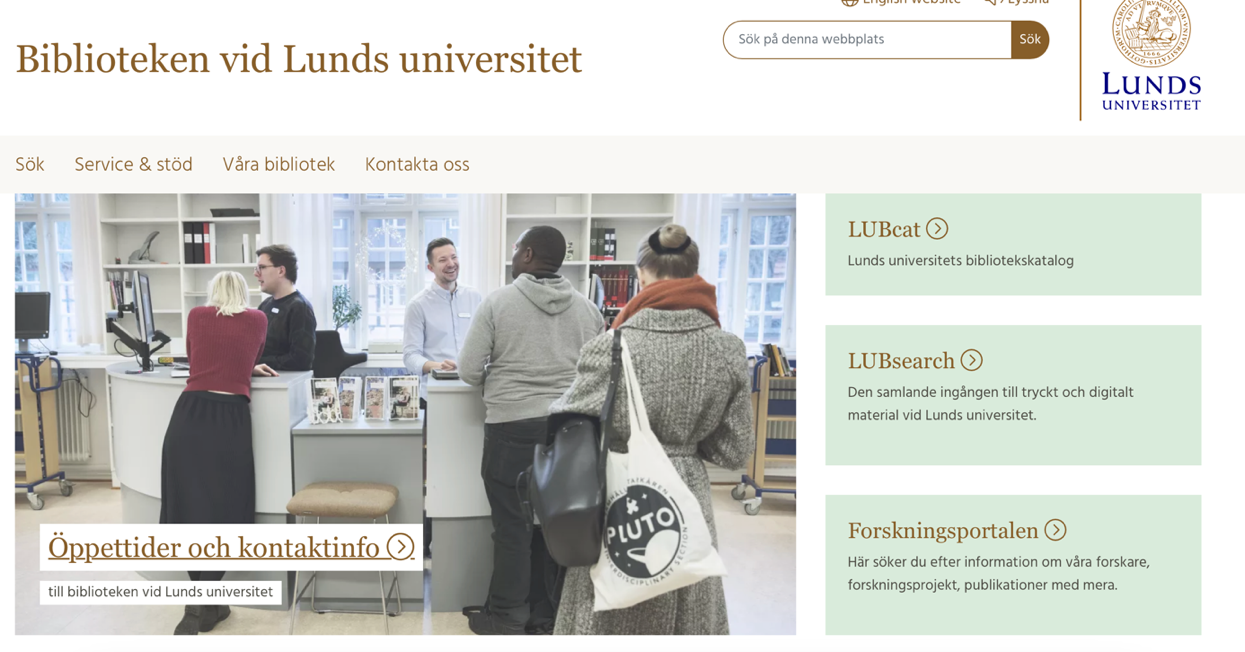


Question 7 (max 1 p)

What does Norman mean by “knowledge in the world” (in relation to “knowledge in the head”)?

Question 8 (max 2 p)

Below are examples of different types of errors (mistakes/slips). Check whether the error is a mistake or a slip.

| Handling | Mistake | Slip |
| --- | --- | --- |
| You’re in a hurry to work but need to refuel on the way. In the stress, you forget to put the gas cap back on. |  |  |
| You think a heart icon in an app means you´ve saved something for later, but it actually means you like it and others can see it. |  |  |
| You try to pull a door that should slide sideways because there are no clear visual cues. |  |  |
| You try to open a link in a browser but accidentally double-click, causing unexpected behavior. |  |  |

Question 9 (max 2 p)

When using Google, links to pages you’ve already visited appear in a different color. What are the advantages of this solution?

Question 10 (max 5 p)

Below is an example from an elevator. Evaluate the design using Norman’s book/concepts and other cognitive science terms you find relevant.

Remember to reason and explain why something is, for example, “poor feedback.”


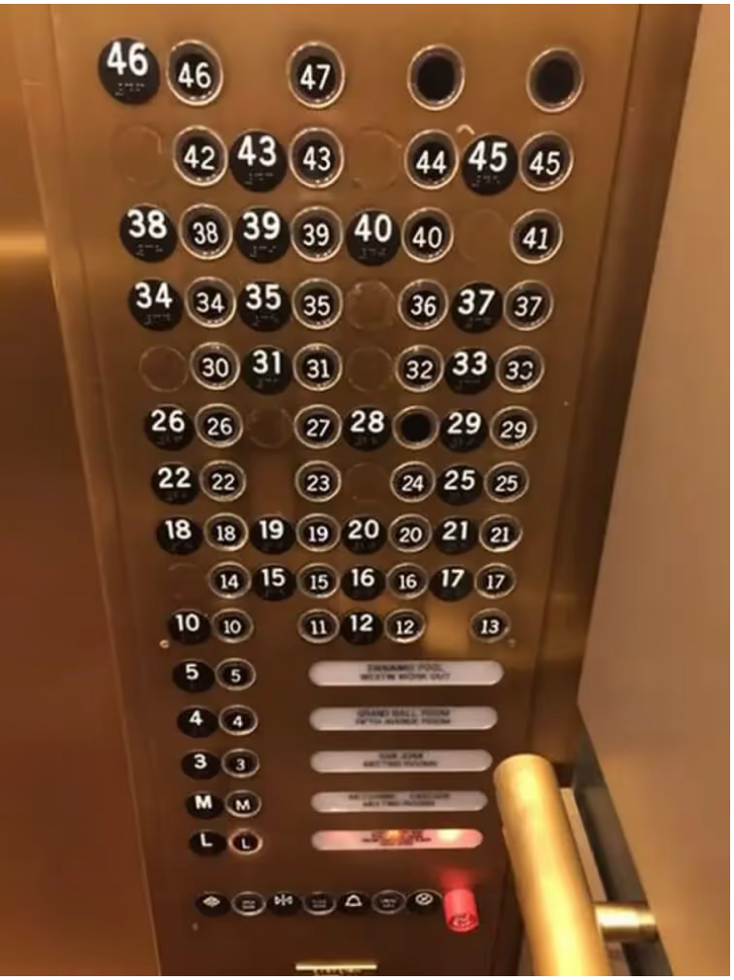


Write clearly. Illegible answers will not be considered.

Good luck!

/Blinded och Blinded

# Appendix E

**Individual assignment EXTA65**

**Assignment**

*Preparation*: Download the Heart-Lung Foundation’s app *Save the Heart* (*Rädda hjärtat*) on your mobile phone (available for both Android and iOS).

*Scenario*: Imagine you are having a supervision meeting with a senior teacher in the program. During the meeting, the teacher has been somewhat short of breath and appeared pale and clammy. Suddenly, the teacher collapses, and you and your classmates cannot get the teacher to wake up. You then notice that the teacher is not breathing. One person runs off to get help, but that may take time. Suddenly, you remember that you were required to download the *Save the Heart* app as part of the Cognition course. You open the app and try to follow the instructions—including attempting to locate a defibrillator that you can use before help arrives.

**Part 1**

Go through the app and try to locate a defibrillator using it. IMPORTANT*: Do not touch the defibrilliator once you have found it.* Make sure you actually complete the step of trying to find the nearest defibrillator, this will help you write a good answer.

*Save the heart – find a defibrillator Save the heart, CPR*

*Do not touch it once you have found it! (studied in part 2)*


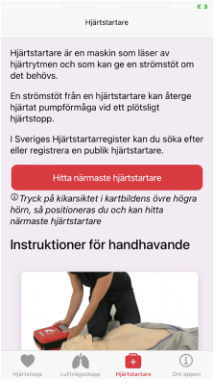

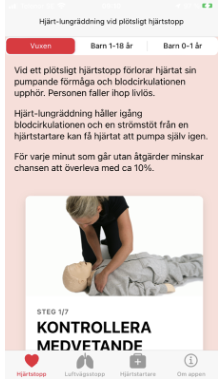


**1.1** (*6 points*) Describe the app’s functionality from a user perspective. Although in reality the most important thing is to start CPR immediately, in this assignment you should focus on how you can use the app to find a defibrillator. Pay special attention to the frightening situation the user is in. Remember that we become less capable when stressed and scared. Focus on whether the app and its instructions follow Norman’s (2013) design recommendations, such as the concepts he uses to discuss usability. Do not start your text with a list of definitions; instead, introduce relevant concepts when you need them to highlight problems or good aspects of the app. You should also discuss potential problems with recognizing the physical defibrillator you located using the app. Feel free to include a photo if you find it appropriate. Use relevant concepts from Norman as described above. *Again: The task is about finding and identifying the defibrillator, not using it. Therefore, do not touch it once you have found it—we want all defibrillators to remain in perfect condition for real emergencies*.

**1.2** (*1 point*) Reasonably, all adults in the population should be able to find a defibrillator using an app like *Save the Heart*. Consider whether there is any subgroup within this broad target audience that should be prioritized in the design, and if so, why. Remember to justify your answer.

**1.3** (*5 points*) Propose changes to the app’s interface and to the appearance and placement of the defibrillator to make it easier for users to find one (again, do not touch it). Address both the app’s visual design, its interactivity, and how the location where the defibrillator hangs should look. Include sketches if possible and explain your reasoning clearly so the reader understands your ideas. Explain and justify your changes using the terminology found in Norman (2013). Do not forget to provide proper references and page numbers.

**1.4** (*3 points*) During the course, you have learned that human attention is limited and guided by expectations. Under stress, this is even more true. Discuss how limited attention could worsen some of the problems you observed with the app or the physical defibrillator, and explain why your solution is better at capturing attention under these special circumstances. Use relevant course literature, such as section 4.5 in *Introduction to Psychology*, Mullet & Sano (1995), or Carpenter (2001).

**1.5** (*mandatory*) Include a brief summary (at least five points) of the most important feedback you received from your peers during peer review and what you did to address this feedback.


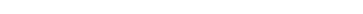


**Part 2**

**(***Optional for grade 4 or 5*)

In this task, you should focus on the instructions for performing CPR. Although the Heart-Lung Foundation states that the app is not intended for use in emergency situations, such a situation could very well occur. Consider how the instructions could be improved to make them easier to follow in a real emergency and provide concrete suggestions for changes. If you want to learn more about CPR, you can check here: <https://www.hlr.nu/utbildningsfilmer/>

**2.1** Describe what you consider to be the biggest problems with the CPR instructions and use relevant terminology. Refer to Norman (2013) as well as what you know about how our visual system and attention work, how we remember, and our ability to interpret information using prior knowledge. Make sure to reference different parts of the course literature (several articles and the course book), and do not forget page numbers (or section names if page numbers are missing, as in *Introduction to Psychology*).

**2.2** Suggest how the instructions, interface, or interactivity could be changed to make them more usable in an emergency. Pay special attention to the fact that the user is likely stressed, will have difficulty thinking in multiple steps, will be clumsy, sweaty (how well does a touchscreen work then?), and easily lose focus. Explain why your suggestions will make things easier for the stressed user. Justify your suggestions and answers using all relevant course literature.
